# Compound 13 activates AMPK-Nrf2 signaling to protect neuronal cells from oxygen glucose deprivation-reoxygenation

**DOI:** 10.18632/aging.102534

**Published:** 2019-12-18

**Authors:** Yanqing Mo, Jian-liang Zhu, Aihua Jiang, Jing Zhao, Liping Ye, Bin Han

**Affiliations:** 1 Minhang Hospital, Fudan University, Minhang District, Shanghai, China; 2 Department of Emergency and Intensive Care Unit, The Second Affiliated Hospital of Soochow University, Suzhou, China

**Keywords:** ischemia-reperfusion, oxidative stress, neuronal cells, AMPK, compound 13

## Abstract

Oxygen glucose deprivation-reoxygenation (OGD-R) causes the production of reactive oxygen species (ROS) and oxidative injury in neuronal cells. We tested the potential neuroprotective function of compound 13 (C13), a novel AMP-activated protein kinase (AMPK) activator, against OGD-R. We show that C13 pretreatment protected SH-SY5Y neuronal cells and primary hippocampal neurons from OGD-R. C13 activated AMPK signaling in SH-SY5Y cells and primary neurons. It significantly inhibited OGD-R-induced apoptosis activation in neuronal cells. Conversely, AMPKα1 shRNA or knockout reversed C13-mediated neuroprotection against OGD-R. C13 potently inhibited OGD-R-induced ROS production and oxidative stress in SH-SY5Y cells and primary neurons. Furthermore, C13 induced Keap1 downregulation and Nrf2 activation, causing Nrf2 stabilization, nuclear accumulation, and expression of Nrf2-dependent genes. Nrf2 silencing or knockout in SH-SY5Y cells abolished C13-mediated neuroprotection against OGD-R. In conclusion, C13 activates AMPK-Nrf2 signaling to protect neuronal cells from OGD-R.

## INTRODUCTION

In the pathogenesis of stroke, ischemia-reperfusion leads to significant oxidative injury, causing severe damage to neurons [1, 2]. In cultured neuronal cells, ischemia-reperfusion is mimicked by oxygen glucose deprivation (OGD)-reoxygenation (OGD-R) [3–6]. OGD-R induces the production of reactive oxygen species (ROS), resulting in neuronal cell death and apoptosis [5, 7]. Conversely, ROS inhibition (*i.e.,* by adding antioxidants) can protect neuronal cells from OGD-R [5, 7].

AMP-activated protein kinase (AMPK) is a master energy sensor. Its activation is vital for energy and metabolism homeostasis [8]. AMPK is primarily composed of the catalytic α subunit along with the regulatory β and γ subunits [9, 10]. Recent studies have shown that forced activation of AMPK, by genetic and/or pharmacological methods, will promote cell survival under different stress conditions [11]. The pro-survival function of AMPK is achieved through regulation of AMPK’s downstream effectors.

AMPK activation can trigger cytoprotective autophagy by directly phosphorylating autophagy-associated proteins, including Unc-51 like autophagy activating kinase1 (Ulk1), Beclin-1, and Vps34 [12, 13]. Furthermore, activated AMPK exerts anti-oxidative effects by inhibiting nicotinamide adenine dinucleotide phosphate (NADPH) consumption. Crosstalk between AMPK and nuclear factor erythroid 2-related factor 2 (Nrf2, a key endogenous anti-oxidant cascade) can also inhibit oxidative injury [14, 15]. Moreover, AMPK can inhibit mammalian target of rapamycin (mTOR), offering a pro-survival outcome under an energy crisis [16, 17].

Recent studies have developed the nucleotide mimetic compound 2 (C2) [18], and its cell-permeable pro-drug compound 13 (C13) [19]. C13 is a novel, potent and α1-selective AMPK activator [19]. Studies have shown that C13 potently inhibits lipid synthesis. It is, however, ineffective in AMPKα1-depleted cells [19]. In the present study, we show that C13 activates AMPK signaling to attenuate OGD-R-induced oxidative injury in neuronal cells.

## RESULTS

### C13 protected SH-SY5Y neuronal cells and primary neurons against OGD-R

**Figure 1:**
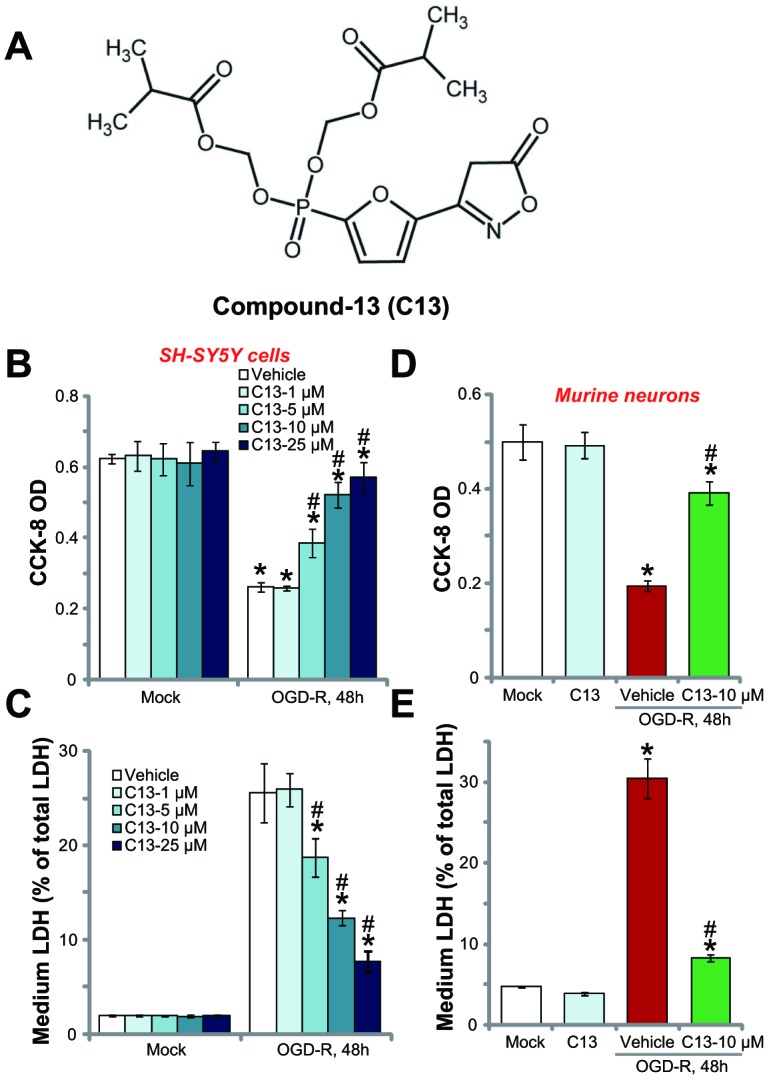
**Compound 13 protected SH-SY5Y neuronal cells and primary neurons from OGD-R.** The structure of C13 is shown in (**A**). SH-SY5Y human neuronal cells (**B** and **C**) or the primary murine hippocampal neurons (**D** and **E**) were treated with applied concentration (1–25 μM) of compound 13 (“C13”), together with/out OGD exposure for 4h, followed by 48h re-oxygenation (“OGD-R”), cell viability was tested by CCK-8 assay (**B** and **D**); Cell death was examined by medium LDH release assay (**C** and **E**). “OGD-R” stands for OGD/re-oxygenation (Same for all figures). “Mock” stands for Mock control cells (no OGD-R, same for all Figures). “Vehicle” stands for the vehicle for C13 (0.1% DMSO, same for all Figures). For the OGD-R experiments, neuronal cells were always pretreated with C13 for 2h before OGD (Same for all figures). Bars stands for mean ± standard deviation (SD, n=5). * *p*<0.05 *vs.* “Mock” cells. ^#^ *p*<0.05 *vs.* cells with “OGD-R” treatment (no C13 pretreatment). Each experiment was repeated four times with similar results obtained.

The structure of C13 is shown in Figure 1A. First, SH-SY5Y cells were subjected to OGD-R (OGD for 4h, followed by reoxidation for 48h). In line with other reports [3, 20, 21], OGD-R led to more than 60% reduction in cell viability (CCK-8 OD) in SH-SY5Y cells (Figure 1B). Significantly, pretreatment of C13 (2h pretreatment) efficiently inhibited the reduction in OGD-R-induced SH-SY5Y cell viability (Figure 1B). The AMPK activator displayed dose-dependent activity in protecting SH-SY5Y cells from OGD-R (Figure 1B). However, it was ineffective at the lowest concentration (1 μM) (Figure 1B). Assaying of cell death by measuring medium LDH release further demonstrated that OGD-R induced significant SH-SY5Y cell death (Figure 1C), and this effect was significantly inhibited by pretreatment with C13 (5–25 μM; Figure 1C). Importantly, C13 single treatment, at the tested concentrations (1–25 μM) did not change SH-SY5Y cell survival (Figure 1B) and cell death (Figure 1C). Therefore, C13 pretreatment protected SH-SY5Y neuronal cells from OGD-R.

The potential effect of C13 in the primary neurons was tested. As shown, OGD-R exposure similarly induced a reduction in CCK-8 OD (Figure 1D) and LDH release (Figure 1E) in primary murine hippocampal neurons. Pretreatment for 2h with the AMPK activator C13 (10 μM) potently inhibited the reduction in viability (Figure 1D) and death (Figure 1E) of primary neurons. C13 treatment by itself was ineffective (Figure 1D and 1E). Therefore, C13 is neuroprotective against OGD-R.

### C13 inhibited OGD-R-induced apoptosis activation in SH-SY5Y neuronal cells and primary neurons

**Figure 2:**
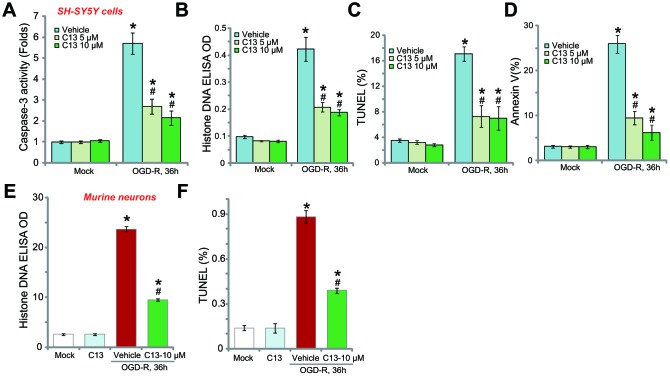
**Compound 13 inhibited OGD-R-induced apoptosis activation in SH-SY5Y neuronal cells and primary neurons.** SH-SY5Y human neuronal cells (**A**–**D**) or the primary murine hippocampal neurons (**E** and **F**) were pretreated (for 2h) with applied concentration (5/10 μM) of compound 13 (“C13”), followed by OGD-R stimulation for applied time periods, cell apoptosis was tested by the assays mentioned in the text. Bars stands for mean ± standard deviation (SD, n=5). * *p*<0.05 *vs.* “Mock” cells. ^#^ *p*<0.05 *vs.* cells with “OGD-R” treatment (no C13 pretreatment). Each experiment was repeated four times with similar results obtained.

The potential effect of C13 on the neuronal cell apoptosis was studied. First, we show that OGD-R induced caspase-3 activation in SH-SY5Y neuronal cells, and this was largely inhibited by pretreatment with C13 (5 or 10 μM, 2h; Figure 2A). The accumulation of histone-bound single strand DNA (ssDNA) is a characteristic marker of cell apoptosis. In OGD-R-treated SH-SY5Y neuronal cells, the amount of histone-bound ssDNA was significantly increased (Figure 2A), and again inhibited by C13 pretreatment (Figure 2B). Furthermore, OGD-R increased the ratios of TUNEL-positive nuclei (Figure 2C) and Annexin V-positive cells (Figure2D), and these effects were also attenuated by C13 (Figure 2C–2D).

In primary murine hippocampal neurons, OGD-R exposure induced the accumulation of histone-bound ssDNA (Figure 2E) and increased the ratio of TUNEL-positive nuclei (Figure 2F), indicating the activation of apoptosis. Pretreatment with C13 (10 μM) potently inhibited the activation of OGD-R-induced apoptosis in the primary neurons (Figure 2E and 2F). C13 treatment alone had no significant effect on apoptosis in SH-SY5Y cells (Figure 2A–2D) and primary neurons (Figure 2E and 2F).

### C13 activates AMPK signaling in SH-SY5Y neuronal cells and primary neurons

**Figure 3:**
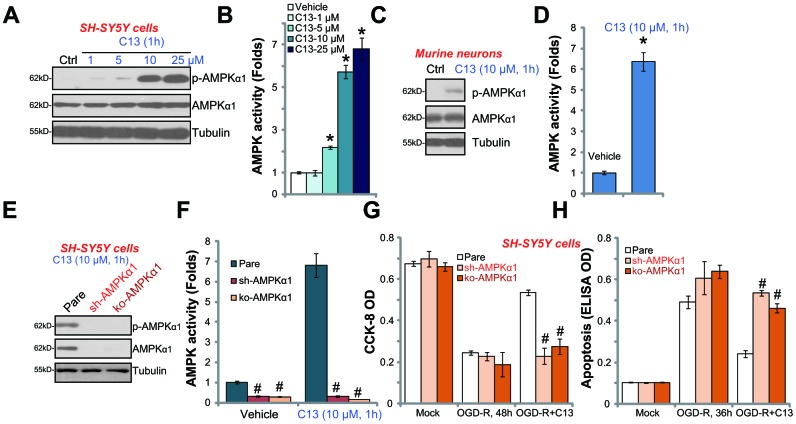
**C13 activated AMPK signaling in SH-SY5Y neuronal cells and primary neurons.** SH-SY5Y human neuronal cells (**A**–**B**) or the primary murine hippocampal neurons (**C** and **D**) were treated with applied concentration of compound 13 (“C13”) for 1h, expression of listed proteins in total cell lysates were shown (**A** and **C**); The AMPK activities were also tested (B and D). The stable SH-SY5Y cells with AMPKα1 shRNA (“sh-AMPKα1”) and CRISPR/Cas-9 AMPKα1-KO construct (“ko-AMPKα1”), as well as the parental control cells (“Pare”), were treated with C13 (10 μM) for 1h, expression of listed proteins (**E**) and AMPK activities (**F**) were tested. The cells were pretreated with C13 (10 μM) for 2h, followed by OGD-R stimulation for applied time periods, cell viability and apoptosis were tested by CCK-8 assay (**G**) and Histone DNA ELISA assay (**H**), respectively. Bars stands for mean ± standard deviation (SD, n=5). * *p*<0.05 *vs.* “Mock” cells. ^#^ *p*<0.05 *vs.* parental control cells. Each experiment was repeated four times with similar results obtained.

C13 is an AMPK activator, we therefore analyzed AMPK signaling in C13-treated neuronal cells. As shown in Figure 3A, in SH-SY5Y cells, C13 dose-dependently increased the phosphorylation of AMPKα1 (at the Thr-172 residue), and the total level of AMPKα1 was unchanged. By using the AMPK activity assay, we show that C13 (5–25 μM) treatment potently increased the activity of AMPK in SH-SY5Y cells (Figure 3B). Thus, C13 activated AMPK signaling in SH-SY5Y cells. In the primary murine hippocampal neurons, the phosphorylation of AMPKα1 (Figure 3C) and the activity of AMPK (Figure 3D) were significantly increased following treatment with C13 (10 μM, 1h).

To block AMPK activation, AMPKα1 shRNA lentivirus was added to SH-SY5Y cells, and stable cells were established via selection through puromycin (“sh-AMPKα1” cells). Additionally, the CRISPR/Cas9 AMPKα1-KO construct (with GFP) was transfected to SH-SY5Y cells. FACS assay-mediated GFP sorting and puromycin selection were employed to establish stable AMPKα1-KO cells (“ko-AMPKα1” cells). Western blotting assay results confirmed that AMPKα1 expression levels were significantly reduced in “sh-AMPKα1” and “ko-AMPKα1” cells (Figure 3E). Consequently, C13 (10 μM, 1h)-induced AMPKα1 phosphorylation (Figure 3E) and AMPK activation (Figure 3F) were completely blocked. Notably, in “sh-AMPKα1” cells and “ko-AMPKα1” cells, C13 was unable to protect the SH-SY5Y cells from OGD-R (Figure 3G and 3H). OGD-R-induced reduction of viability and apoptosis in SH-SY5Y cells were not significantly inhibited by C13 when AMPKα1 was silenced or depleted (Figure 3G and 3H).

### C13 inhibited OGD-R-induced oxidative stress in neuronal cells

**Figure 4:**
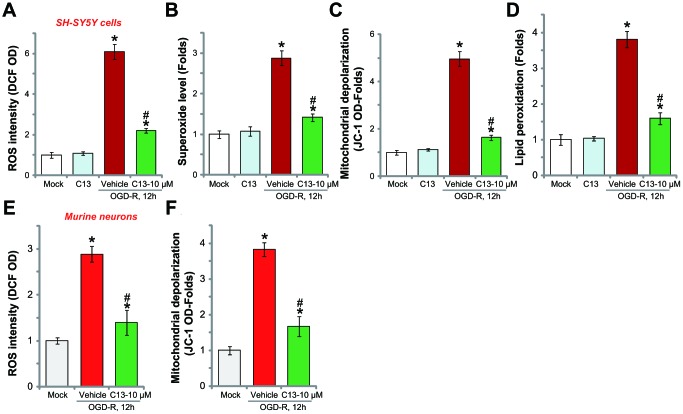
**C13 inhibited OGD-R-induced oxidative stress in neuronal cells.** SH-SY5Y human neuronal cells (**A**–**D**) or the primary murine hippocampal neurons (**E** and **F**) were pretreated with C13 (10 μM) for 2h, followed by OGD-R stimulation for applied time, ROS contents were tested by the appropriate assays (A, B and E); Mitochondrial depolarization and lipid peroxidation levels were tested by JC-1 dye assay (**C** and **F**) and TBAR activity assay (**D**), respectively. Bars stands for mean ± standard deviation (SD, n=5). * *p*<0.05 *vs.* “Mock” cells. ^#^ *p*<0.05 *vs.* cells with “OGD-R” treatment (no C13 pretreatment). Each experiment was repeated three times with similar results obtained.

OGD-R stimulation in neuronal cells induces profound ROS production and oxidative stress, causing cell death and apoptosis [20, 21]. Recent studies have shown that forced-activation of AMPK could induce potent antioxidant activity [22–24]. In SH-SY5Y cells, OGD-R treatment significantly increased the DCF-DA intensity (Figure 4A) and superoxide contents (Figure 4B), indicating ROS production. Furthermore, mitochondrial depolarization (JC-1 intensity increase, Figure 4C) and lipid peroxidation (TBAR intensity increase, Figure 4D) were detected, further indicating oxidative injury. Significantly, C13 pretreatment (10 μM, 2h) potently inhibited OGD-R-induced ROS production (Figure 4A and 4B) and oxidative injury (Figure 4C and 4D) in SH-SY5Y cells, and C13 single treatment was ineffective (Figure 4A–4D). In the primary murine hippocampal neurons, OGD-R-induced ROS production (tested by the DCF-DA intensity increase, Figure 4E) and mitochondrial depolarization (JC-1 intensity increase, Figure 4F) were also attenuated by C13 pretreatment. These results show that C13 inhibited OGD-R-induced oxidative stress in neuronal cells.

### C13 activated AMPK downstream Nrf2 signaling in neuronal cells

Nrf2 signaling is the key endogenous antioxidant signaling cascade [25, 26]. Once activated, Nrf2 will separate from Keap1, causing Nrf2 protein stabilization, accumulation, and activation [25, 26]. Activated Nrf2 translocates to and accumulates in cell nuclei where it binds to ARE (antioxidant response element) to initiate transcription and the expression of antioxidant genes, including *HO1*, *NQO1,* and *GCLC* [25, 26]. Existing studies have established that there is a crosstalk between AMPK and Nrf2 signaling [14, 15, 27]. Therefore, we tested whether C13 could also activate Nrf2 signaling in neuronal cells.

**Figure 5:**
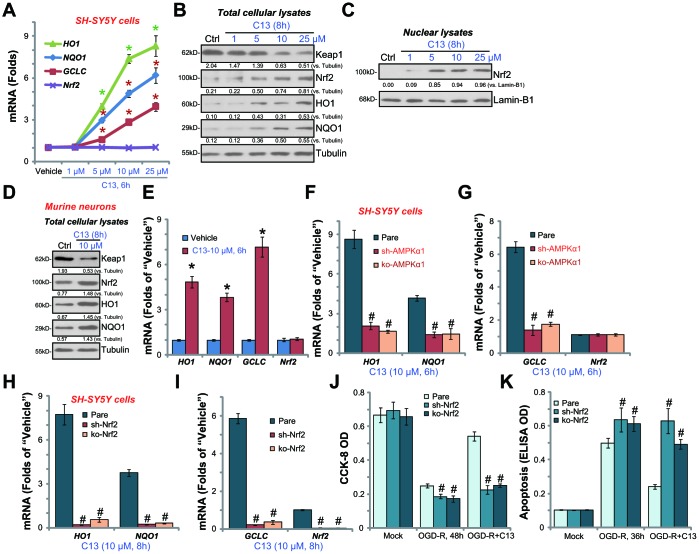
**C13 activated AMPK downstream Nrf2 signaling in neuronal cells.** SH-SY5Y human neuronal cells (**A**–**C**) or the primary murine hippocampal neurons (**D** and **E**) were treated with applied concentration of C13 for indicated time, expression of listed mRNAs (**A** and **E**) and proteins (in both total lysates and nuclear fraction lysates, **B**–**D**) were shown. The stable SH-SY5Y cells with AMPKα1 shRNA (“sh-AMPKα1”) and CRISPR/Cas-9 AMPKα1-KO construct (“ko-AMPKα1”), as well as the parental control cells (“Pare”), were treated with C13 (10 μM) for indicated time, expression of listed mRNAs were shown (**F** and **G**). The stable SH-SY5Y cells with Nrf2 shRNA (“sh-Nrf2” cells) and CRISPR/Cas-9 Nrf2-KO construct (“ko-Nrf2” cells), as well as the parental control cells (“Pare”), were treated with C13 (10 μM) for indicated time, listed mRNAs were shown (**H** and **I**); Cells were also pretreated with C13 (10 μM) for 2h, followed by OGD-R stimulation for 36/48h, cell viability (CCK-8 assay, **J**) and apoptosis (Histone DNA ELISA assay, **K**) were tested. Expression of listed proteins were quantified and normalized to the loading controls (**B**–**D**). Bars stands for mean ± standard deviation (SD, n=5). * *p*<0.05 *vs.* “Vehicle” control cells (**A** and **E**). ^#^ *p*<0.05 *vs.* “Pare” cells (F-K). Each experiment was repeated three times with similar results obtained.

By employing quantitative real-time PCR (qPCR), we show that *HO1*, *NQO1,* and *GCLC* mRNA levels were significantly increased in C13 (5-25 μM)-treated SH-SY5Y cells (Figure 5A). *Nrf2 mRNA* level was unchanged (Figure 5A) but Nrf2 protein, along with HO1, NQO1, and GCLC were significantly increased following C13 (5-25 μM) treatment (Figure 5B). Importantly, Keap1, the negative regulator of Nrf2, was downregulated with C13 treatment in SH-SY5Y cells (Figure 5B). By analyzing nuclear fraction lysates, we show that Nrf2 translocated to cell nuclei following C13 treatment (Figure 5C). In the primary murine hippocampal neurons, C13 (10 μM) similarly induced Keap1 downregulation, Nrf2 protein stabilization, as well as protein and mRNA expression of *HO1*, *NQO1,* and *GCLC* (Figure 5D and 5E).

The results above show that C13 activated Nrf2 signaling in SH-SY5Y cells and primary murine hippocampal neurons (Figure 5A–5E). Importantly, in Figure 5F and 5G, C13-induced mRNA expression of *HO1*, *NQO1,* and *GCLC* was almost completely blocked in “sh-AMPKα1” and “ko-AMPKα1” cells (see Figure 3). These results imply that activation of AMPK mediates the activation of C13-induced Nrf2 signaling in SH-SY5Y cells.

To test the link between Nrf2 activation and C13-mediated neuroprotection against OGD-R, shRNA strategy and CRISPR/Cas9 method were employed to silence and knockout Nrf2, respectively. In the Nrf2-silenced (“sh-Nrf2”) or Nrf2-KO (“ko-Nrf2”) SH-SY5Y cells, C13 (10 μM)-induced *HO1*, *NQO1,* and *GCLC* mRNA expression was blocked (Figure 5H and 5I). Significantly, in Nrf2-silenced or Nrf2-KO SH-SY5Y cells, the addition of C13 failed to protect SH-SY5Y cells from OGD-R (Figure 5J and 5K). Therefore, Nrf2 signaling activation is a key downstream of AMPK that mediates C13-induced neuroprotection against OGD-R.

## DISCUSSION

AMPK participates in a number of key cellular behaviors, from energy metabolism, cell mitosis, cell survival, and cell growth to apoptosis and autophagy [13, 28]. Studies have demonstrated that AMPK activation could promote cell survival under stress conditions [29, 30]. AMPK is reported to be overactivated in the brains of patients with neurodegenerative disorders, including Alzheimer's disease, Parkinson's disease, Huntington's disease, and amyotrophic lateral sclerosis [31, 32]. The exact function of AMPK in neurological diseases remains to be clarified, but existing studies have reported the involvement of AMPK in various signaling pathways that are important for the survival of neuronal cells [31, 32]. Here, we show that the activation of AMPK by C13 protected SH-SY5Y cells and primary hippocampal neurons from OGD-R. Conversely, AMPKα1 shRNA or KO reversed C13-mediated neuroprotection. Thus, C13-induced AMPK activation offers significant protection to neuronal cells against OGD-R.

Studies have reported on the potent antioxidant activity mediated by activated AMPK [30]. In addition to its role in ATP homeostasis, forced activation of AMPK could attenuate oxidative injury by promoting NADPH synthesis and/or inhibiting NADPH consumption [30]. Studies have also established that there is a crosstalk between AMPK and Nrf2 signaling cascades [14, 15, 33]. Joo et al*.,* show that AMPK directly phosphorylates Nrf2 at Serine 550 to promote Nrf2 nuclear translocation and activation [14]. The results of the present study show that C13 activated AMPK downstream Nrf2 signaling to inhibit OGD-R-induced ROS production and oxidative stress, thus protecting neuronal cells from OGD-R. Importantly, we proposed a novel mechanism for AMPK-induced Nrf2 signaling activation, that is, downregulation of Keap1.

Under resting conditions, Nrf2 binds to Keap1, causing Cul3-dependent Nrf2 ubiquitination and proteasomal degradation. We showed that C13 downregulated Keap1 and caused Nrf2 accumulation and activation of Nrf2 cascade; the latter was evidenced by Nrf2 protein stabilization, nuclear accumulation, and expression of Nrf2-depenedent genes (*HO1*, *NQO1,* and *GCLC*) in neuronal cells. Significantly, Nrf2 shRNA or KO in SH-SY5Y cells almost abolished C13-mediated neuroprotection against OGD-R. These results suggest that Nrf2 activation, downstream of AMPK, mediates C13-induced neuroprotection from OGD-R-induced oxidative injury. Indeed, we show that C13 potently inhibited OGD-R-induced ROS production, mitochondrial depolarization, and lipid peroxidation in SH-SY5Y cells and primary neurons. The underlying mechanisms of C13-induced Nrf2 signaling activation needs further investigation.

The present study has shown that C13 activated AMPK-Nrf2 signaling to protect the neuronal cells from OGD-R, a cellular model of ischemic stroke. It would be interesting to further test the potential of this compound against ischemic stroke.

## METHODS

### Reagents

C13 was provided by Dr. Wang [34]. Puromycin, polybrene, the Annexin V-FACS assay kit, the caspase-3 assay kit, and cell culture reagents were purchased from Sigma-Aldrich (St. Louis, MO). Antibodies were obtained from Santa Cruz Biotechnology (Santa Cruz, CA) and Cell Signaling Technology (Shanghai, China). Terminal deoxynucleotidyl transferase dUTP nick end labeling (TUNEL), JC-1, and carboxy-H2DCFDA dyes were purchased from Invitrogen ThermoFisher Scientific (San Jose, CA). The Histone DNA enzyme-linked immunosorbent assay (ELISA) kit was provided by Roche (Shanghai, China).

### SH-SY5Y cells

The human neuroblastoma cell line SH-SY5Y was purchased from the Cell Bank of Fudan University (Shanghai, China). SH-SY5Y cells were cultured in Dulbecco’s modified Eagle’s medium (DMEM) with 10% fetal bovine serum and penicillin/streptomycin (1:100, Sigma-Aldrich). For neuronal cell differentiation, SH-SY5Y cells were cultured for seven days in serum-free DMEM medium with brain-derived neurotrophic factor and glutamine. Only differentiated SH-SY5Y cells were subjected to the OGD-R procedure.

### Murine hippocampal neurons

Cultures of primary murine hippocampal neurons have been reported previously [35]. Briefly, primary neurons of CA1 hippocampus of E12-E14 embryos of C57 mice were separated and the CA1 hippocampal neurons were plated onto serum-free neuron basal medium with 2% B27 supplement and 2 mM glutamine. At day-8 *in vitro*, neurons were subjected to the OGD-R procedure.

### OGD-R

The detailed procedures of OGD-R have been previously described [3]. Briefly, neuronal cells were placed in an airtight chamber and equilibrated for 10-12 min with a continuous flux of gas (95% N_2_and 5% CO_2_). The chamber was sealed and placed in an incubator for 4h under OGD. Cells were then reoxygenated. “Mock” cells were placed in DMEM containing glucose under normal oxygenation.

### Cell survival assay

The Cell Counting Kit-8 (CCK-8) assay (Dojindo Laboratories, Kumamoto, Japan) was employed to test cell viability, with its optical density (OD) values recorded at a wavelength of 450 nm.

### Cell death assay

Cell death was examined by measuring lactate dehydrogenase (LDH) medium release via a two-step easy enzymatic reaction LDH assay kit (Takara, Tokyo, Japan). The LDH content in medium was normalized to the total LDH content.

### Apoptosis assays

Routine apoptosis assays, including caspase-3 activity assay, Histone DNA ELISA assay, TUNEL staining assay, and the Annexin V FACS assay have been described in detail in previous studies [36–38].

### Western blotting assay

The RIPA lysis buffer (Biyuntian Co., Wuxi, China) was added to neuronal cells. After quantification, 30 μg of lysate proteins per treatment was separated by 10-12.5% sodium dodecyl sulfate-polyacrylamide gel electrophoresis and transferred to polyvinylidene fluoride blots (Merck Millipore, Darmstadt, Germany). After blocking in 10% non-fat milk in phosphate-buffered saline with Tween 20 (PBST), the blots were incubated with the appropriate primary antibodies. After three washes with PBST, the secondary antibodies were added and detected using an enhanced chemiluminescence system (Amersham, Little Chalfont, UK). The intensity (total gray area) of each band was quantified via using ImageJ software (National Institutes of Health, Bethesda, MD), and the value was normalized to the corresponding loading control.

### ROS assay

As previously described [21], ROS contents were tested by the carboxy-H2DCFDA dye assay. Following the applied treatment, neuronal cells were stained with carboxy-H2-DCFDA (5 μM) for 30 min in the dark. DCF fluorescence was tested by a fluorescence spectrofluorometer (Thermo Fisher Scientific) at 485 nm excitation and 525 nm emission.

### Superoxide assay

A superoxide assay kit (Beyotime, Wuhan, China) was utilized to examine the cellular superoxide contents based on the attached protocol. Briefly, neuronal cells with the applied treatments were incubated with the superoxide detection reagent (100 μL/well) for 30 min in the dark at room temperature. Superoxide absorbance was tested by a spectrophotometer at a wavelength of 450 nm.

### Lipid peroxidation assay

Following the applied treatment, thiobarbituric acid reactive substances (TBAR) activities were examined to quantify the cellular lipid peroxidation levels using a reported protocol [21, 39].

### Mitochondrial depolarization assay

As previously described [40], in mitochondrial depolarization, the JC-1 red fluorescence aggregates to form green monomers [41]. Briefly, after the applied treatment, neuronal cells were stained with JC-1 (5 μg/mL) for 20 min in the dark at room temperature. After three washes, JC-1 green intensity was examined immediately at 550 nm via a fluorescence spectrofluorometer.

### Quantitative real-time PCR assay

Cellular RNA was extracted via TRIzol reagents (Promega, Madison, WI). For quantitative real-time PCR, a SYBR Green PCR kit (Applied Biosystems, Foster City, CA) was utilized for reverse transcription under the ABI Prism7600 Fast Real-Time PCR system. The primers of *human GCLC*, *Nrf2*, *HO1*, *NQO1,* and *GAPDH* have been described previously [42]. Primers of *murine GCLC*, *Nrf2*, *HO1*, *NQO1*, and *GAPDH* have also been described [43].

### AMPKα1or Nrf2 short hairpin RNA

Lentiviral particles, encoding AMPKα1 short hairpin RNA (shRNA;sc-44281-V), Nrf2 shRNA (sc-44332-V) or control shRNA (sc-108080) were purchased from Santa Cruz Biotechnology. SH-SY5Y cells were plated onto six-well plates at a density of 2 × 10^5^ cells/mL in the polybrene-containing complete medium. Lentivirus particles were added to SH-SY5Y cells for 12h. Puromycin (3.0 μg/mL) was then added to select stable cells for a total of six passages (10-12 days). AMPKα1or Nrf2 silencing in stable cells was tested by western blotting.

### CRISPR/Cas9-induced knockout of AMPKα1 or Nrf2

Small guide RNA against human AMPKα1 or human Nrf2 was selected from Optimized Crispr Design software from Dr. Zhang’s laboratory (http://crispr.mit.edu/), and individually inserted into the lentiCRISPR-green fluorescent protein (GFP) plasmid with a puromycin selection gene (Addgene, Watertown, MA). SH-SY5Y cells were plated onto six-well plates at a density of 2 × 10^5^ cells/mL in polybrene-containing complete medium. The lentiCRISPR AMPKα1-knockout (KO) plasmid or the lentiCRISPR Nrf2-KO plasmid was transfected to SH-SY5Y cells by Lipofectamine 2000 reagent. The transfected cells were then subjected to FACS-mediated GFP sorting and selected by puromycin (3.0 μg/mL) for six passages. The resulting stable cells were assayed by western blotting of AMPKα1 or Nrf2 expression. Control cells were transfected with the empty vector.

### Statistical analysis

Data are expressed as the mean ± standard deviation. Statistically significant differences were tested by one-way analysis of variance using Tukey’s post hoc multiple comparisons tests (SPSS version 21.0, SPSS Inc., Chicago, IL). Two group comparisons were performed using Student’s t test (Excel 2007). *P*< 0.05 was considered statistically significant.
